# Baicalein Exerts Neuroprotective Effects in FeCl_3_-Induced Posttraumatic Epileptic Seizures *via* Suppressing Ferroptosis

**DOI:** 10.3389/fphar.2019.00638

**Published:** 2019-06-07

**Authors:** Qin Li, Qiu-Qi Li, Ji-Ning Jia, Qian-Yi Sun, Hong-Hao Zhou, Wei-Lin Jin, Xiao-Yuan Mao

**Affiliations:** ^1^Department of Clinical Pharmacology, Xiangya Hospital, Central South University, Changsha, China; ^2^Institute of Clinical Pharmacology, Central South University, Hunan Key Laboratory of Pharmacogenetics, Changsha, China; ^3^Engineering Research Center of Applied Technology of Pharmacogenomics, Ministry of Education, Changsha, China; ^4^National Clinical Research Center for Geriatric Disorders, Changsha, China; ^5^Centers for Translational Medicine, Ruikang Hospital, Guangxi University of Chinese Medicine, Nanning, China; ^6^Institute of Nano Biomedicine and Engineering, Department of Instrument Science and Engineering, Key Laboratory for Thin Film and Microfabrication Technology of Ministry of Education, School of Electronic Information and Electronic Engineering, Shanghai Jiao Tong University, Shanghai, China; ^7^Shaanxi Key Laboratory of Brain Disorders and Institute of Basic and Translational Medicine, Xi’an Medical University, Xi’an, China

**Keywords:** posttraumatic epileptic seizures, baicalein, 12/15-lipoxygenase, lipid peroxidation, ferroptosis, neuroprotective

## Abstract

Posttraumatic epilepsy (PTE) is a prevalent type of acquired epilepsy secondary to traumatic brain injury, and is characterized by repeated seizures. Traditional antiepileptic drugs have minimal response in preventing posttraumatic epileptic seizures. It is essential for the development of new therapeutic strategy. Our previous work disclosed a potent neuroprotective role of baicalein, a flavonoid extracted from *Scutellaria baicalensis* Georgi, against inherited epilepsy in rats. Whether baicalein has protective potential in posttraumatic epileptic seizures and the possible molecular mechanism remain elusive. Additionally, the brain is vulnerable to lipid peroxidation-induced damage due to high consumption of oxygen and abundant polyunsaturated fatty acids in neuronal membranes. Our present investigation aimed to elucidate whether baicalein exerts neuroprotective effects on posttraumatic epileptic seizures by inhibiting ferroptosis, a newly discovered lipid peroxidation-dependent cell death modality. We found that baicalein significantly reduced seizure score, number of seizures, and average seizure duration in an iron chloride (FeCl_3_)-induced PTE mouse model. The neuroprotective effect of baicalein was also validated in a ferric ammonium citrate (FAC)-induced HT22 hippocampal neuron damage model. Moreover, *in vitro*, baicalein could remarkably decrease ferroptotic indices (lipid reactive oxygen species, 4-hydroxynonenal, and prostaglandin endoperoxide synthase 2) and inhibit the expression of 12/15-lipoxygenase (12/15-LOX) in an iron-induced HT22 cell damage model. These findings were also validated in a mouse PTE model. It was concluded that baicalein exerted neuroprotective effects against posttraumatic epileptic seizures *via* suppressing ferroptosis and 12/15-LOX was likely to be involved in baicalein’s neuroprotection.

## Introduction

Posttraumatic epilepsy (PTE), a recurrent seizure disorder, is the consequence of traumatic brain injury (TBI) (Agrawal et al., 2006; Kharatishvili and Pitkänen, 2010; Lamar et al., 2014; Pitkanen et al., 2014). It is estimated that nearly 80% of patients suffer from seizures within 1 year post-TBI (Volavka, 2011). Epidemiological studies have also shown that more than 3 million people have PTE globally, comprising approximately 5–6% of all epilepsy patients (Pi et al., 2014; Szaflarski et al., 2014; Singh and Trevick, 2016; Chartrain et al., 2017). Additionally, PTE is the most common cause of new onset epilepsy in young adults (Annegers and Coan, 2000). PTE not only significantly reduces the quality of life of patients physically, cognitively, and emotionally, but also increases the risk of mortality (Englander et al., 2009; Christensen, 2015; Xu et al., 2017). Traditional antiepileptic drugs have minimal response in seizures after TBI (Chartrain et al., 2017). Conceptually, the latency between injury and seizures is usually very short. It is hoped that effective treatment could be administered at some point within this latency to stop the process, so that posttraumatic epileptic seizures will not occur. Currently, neuroprotection is increasingly considered as a promising therapy for preventing and treating epilepsy (Acharya et al., 2008; McKinnon et al., 2018). Identification of potential compounds or methods that provide neuroprotective effects on posttraumatic epileptic seizures is important.

Traditional Chinese medicine (TCM) has a long history of preventing and curing diseases (Wu et al., 2011; Mao et al., 2018). Recently, TCM has demonstrated good prospects for improving neurological diseases, cancer, and cardiovascular disease due to its easy penetration of the blood–brain barrier and substantial existence in nature (Chen et al., 2019; Pan and Ma, 2019). TCM possessed various beneficial biological effects, including antioxidant, anti-inflammatory, antitumor, etc. (Li and Zhang, 2008; Pan et al., 2011; Zhang et al., 2019). For example, baicalein, curcumin, and resveratrol exert neuroprotection against neurological diseases, such as epilepsy, ischemic brain damage, and Alzheimer’s disease (AD) (Ahn et al., 2007; Liu et al., 2012; Liu et al., 2015; Lu and Wang, 2015; Gu et al., 2016; Li et al., 2016; Andrade et al., 2018). Particularly, baicalein acted neuroprotective effect in inherited epilepsy (Mao et al., 2014) and in a pilocarpine-induced acute epileptic rats model (Liu et al., 2012) by inhibiting oxidative stress. However, little is known about the neuroprotective effects of baicalein in posttraumatic epileptic seizures. Although it was previously reported that baicalein could protect hippocampal neurons in the PTE model through its antioxidant capacity (Hamada et al., 1993), its specific molecular mechanism remains unclear.

The antioxidant capacity is one of the important potential mechanisms of TCM for neuroprotective effects. For instance, baicalein was reported to protect cerebral ischemia/reperfusion injury *via* inhibiting the function of NF-κB (Liu et al., 2015). And a large body of evidence has demonstrated that higher levels of oxidative stress markers including elevated superoxide dismutase activity, lipid oxidation products, and protein nitrotyrosine exist in the brain of some neurological diseases (Islam, 2017; Poprac et al., 2017; Zhang et al., 2018a). These indicate that oxidative stress is an important target for the neuroprotective effects of many TCM including baicalein. Specifically, due to highly enriched polyunsaturated fatty acids (PUFAs) in the brain, lipid peroxidation is likely to more frequently occur during oxidative damage (Bazinet and Laye, 2014). It has been reported that lipoxygenases (LOXs) serve as a type of key enzymes which involve in the synthesis of lipid hydroperoxide and the facilitation of catalyzing the oxidation of PUFAs (Bazinet and Laye, 2014; Wenzel et al., 2017). As a major subtype of LOX family, 12/15-LOX mediates the oxidation of arachidonic acid (Wenzel et al., 2017) and inhibits the intracellular lipid deposition in foam cells (Belkner et al., 2005), suggesting that it is a key enzyme in lipid peroxidation. Additionally, baicalein was previously found to significantly suppress the expression of 12/15-LOX and protect neuronal cells from death in various neurological diseases such as ischemic brain damage, and AD (van Leyen et al., 2006; Gu et al., 2016). Therefore, our present work aimed to explore whether baicalein could exert neuroprotective effects on (FeCl_3_)-induced posttraumatic epileptic seizures by inhibiting 12/15-LOX-mediated lipid peroxidation. Recently, lipid peroxidation has been found to cause a novel type of cell death, ferroptosis, which constitutes a cell death pathway that is genetically, morphologically, and biochemically different from apoptosis and autophagy (Dixon et al., 2012; Yang and Stockwell, 2016; Conrad et al., 2018). Hence, we also explored whether baicalein could abrogate ferroptosis by 12/15-LOX-mediated lipid peroxidation and finally exert neuroprotective effects on posttraumatic epileptic seizures.

Our data indicated that both baicalein and ferroptosis inhibitors could ameliorate epileptic seizure behavior in FeCl_3_-induced PTE model mice. Furthermore, we found that baicalein could exert neuroprotective effects in FAC-induced HT22 cell damage model and FeCl_3_-induced seizures by suppressing ferroptosis and 12/15-LOX is involved in baicalein’s neuroprotection. We believe that targeting ferroptosis could promote the clinical application of baicalein and might contribute substantially to the prevention of posttraumatic epileptic seizures.

## Materials and Methods

### Chemicals and Reagents

Dulbecco’s modiﬁed Eagle’s medium (DMEM) and fetal bovine serum (FBS) were purchased from GIBCO (Grand Island, NY, USA). Ferric ammonium citrate (FAC) and iron chloride (FeCl_3_) were obtained from Sigma-Aldrich (St. Louis, MO, USA). Baicalein, erastin, ferrostatin-1 (Fer-1), and liproxstatin-1 (Lipo-1) were purchased form Selleck Chemicals (Houston, TX, USA).

### Animals and Establishment of FeCl_3_-Induced PTE Model

All adult male C57/BL6 mice weighing 18–22 g were obtained from the Experimental Animal Center of Central South University, China. The protocol of animal experiment was approved by the Medical Ethics Committee of Xiangya Hospital and performed in accordance with the National Institutes of Health *Guide for the Care and Use of Laboratory Animals*. All efforts had been made to minimize the animal’s suffering. All animals were housed in cages with a 12/12-h light/dark cycle and other standard laboratory conditions, including a room temperature of 23 ± 1°C and access to food and water *ad libitum*. After anesthetization with 10% chloral hydrate (0.3 ml/100 g, i.p.) and stereotaxic placement, the mice were drilled with burr holes in the surface of the skull at stereotaxically marked sites. FeCl_3_ (5 μl containing 50 mM FeCl_3_) dissolved in phosphate buffer saline (PBS) was stereotaxically injected in 5 min through the burr hole (coordinates anteroposterior −1.3 mm; lateral 2.0 mm; and ventral 1.6 mm) in the somatosensory region of the cortex. The needle was kept in place for an additional 10 min after injection to avoid reflux. Animals were randomly divided into six groups as follows: 1) control group (n = 6) was given vehicle intracranial injection (PBS 5 μl); 2) FeCl_3_ group (n = 6) was given 5 μl 50 mM FeCl_3_; 3) and 4) baicalein groups were pretreated with baicalein 50 mg/kg (n = 6) and 100 mg/kg (n = 6) 30 min prior to FeCl_3_ administration, respectively; 5) ferroptosis inhibitor group (n = 6) was administered continuously with 10 mg/kg Lipo-1 3 d prior to FeCl_3_ administration; and 6) baicalein administration group (n = 6) was given 100 mg/kg baicalein once by intraperitoneal injection 30 min prior to 5 μl PBS administration.

### Behavior Tasks

The behavioral changes of mice were recorded within 6 h after injection of FeCl_3_. The scoring system described by Racine was used for evaluating the seizure severity of the treated mice (Racine, 1972; Sato et al., 1990). Racine stages were described as follows: stage 0, no response; stage 1, ear and facial twitching; stage 2, convulsive twitching axially through the body; stage 3, myoclonic jerks and rearing; stage 4, turning over onto the side, wild running, and wild jumping; stage 5, generalized tonic–clonic seizures; and stage 6, death. If the stage 3 seizure or higher were observed, animals were considered to have seizures. Over an observation time of 6 h after FeCl_3_ injection, the number of seizures and the single episode duration of individual seizures were recorded for each mouse simultaneously by two trained observers blinded to the animals’ drug treatment designs.

### Cell Cultures and Drug Treatments

Immortalized mouse hippocampal HT22 cells were cultured in DMEM (C11995500BT, Gibco, USA) containing 10% FBS and 100 units of penicillin and 100 µg/ml streptomycin. Cells were then incubated at 37°C in a 5% CO_2_ atmosphere. For experiments, HT22 cells were pretreated with 12.5 μM Fer-1 (S7243, Selleck, USA) or 1 μM Lipo-1 (S7699, Selleck, USA) for 2 h. Then, 300 μM FAC (F5879, Sigma, USA) was added to the medium for 36 h.

### Cell Viability Assay

Cell viability assays were performed using Cell Counting Kit-8 (CCK8) (C0038, Beyotime Institute of Biotechnology, China) following the manufacturer’s instructions. Briefly, cells were seeded in 96-well plates in a medium containing 10% FBS. After drug treatment at the indicated time points, 10 μl CCK8 solution was added to each well and incubated for 1 h. Cell viability was finally measured using a microplate reader at a wavelength of 450 nm.

### Cell Morphological Observation by Inverted Microscope

HT22 cells were seeded in 24-well plates at a density of 15% and cultured overnight. The plated cells were pretreated with 32 μM baicalein (S2268, Selleck, USA) for 2 h. Then, medium containing FAC (F5879, Sigma, USA) or erastin (S7242, Selleck, USA) at the final concentration of 300 μM and 500 nM were added and cultured for 36 and 8 h, respectively. Alteration of morphology in treated cells was observed under an inverted microscope.

### Detection of Lipid Reactive Oxygen Species (Lipid ROS) by Flow Cytometry

After treatment at the indicated time, cells were washed with PBS, harvested by trypsinization, resuspended in 500 μl Hanks’ balanced salt solution (HBSS) (14065056BT, Gibco, USA) containing 2 μl C11-BODIPY 581/591 (D3861, Thermo Fisher, USA), and incubated for 15 min at 37°C in a tissue culture incubator. Cells were washed with HBSS, centrifuged at 3,000 × *g* for 5 min, and then resuspended in 200 μl PBS. A minimum of 10,000 events per replicate were collected and analyzed using a flow cytometer. Data were collected from the FL1 channel, and subsequently analyzed with FlowJo software.

### Real-Time RT-PCR Analysis

Total RNA was extracted using TRIzol reagent (Invitrogen) according to the manufacturer’s procedure. The extracted RNA was reverse transcribed into cDNA by a reverse transcription kit (Perfect Real Time) (RR047A, Takara Bio, Japan). Real-time PCR was performed using double-stranded DNA dye SYBR Green (RR091A, Takara, Japan) and LightCycler 480 Roche system. PCR conditions were as follows: 30 s hot-start at 95°C followed by 40 cycles of 5 s at 95°C, 30 s at 55°C and 30 s at 72°C; 30 s melting curve at 95°C. All samples were analyzed in triplicate, and gene expression was normalized to β-actin mRNA levels. Primer sequences designed to detect speciﬁc genes are as follows: PTGS2 forward: 5’-GGGAGTCTGGAACAT TGTGAA-3’, reverse: 5’-GTGCACATTGTAAGTAGGTGGACT-3’; ATCB forward: 5’-GTGACGTTGACATCCGTAAAGA-3’, reverse: 5’-GCCGGACTCATCGTACTCC-3’.

### Immunofluorescence

HT22 cells were seeded in a 35-mm dish at a certain density. After drug treatments, cells were fixed in 4% paraformaldehyde for 30 min, permeabilized in 0.2% Triton X-100 for 10 min, and blocked in donkey serum (SL034, Solarbio, China) for 30 min, all at room temperature. Cells were then incubated with primary antibody (12/15-LOX, rabbit, BS8547, 1:100 dilution, Bioworld) in blocking buffer at 4°C overnight. PBS replaced the primary antibody in negative controls. After washing three times with PBS, the cells were then incubated with secondary antibody. The secondary antibody was donkey anti-rabbit Alexa Fluor 488 (711-545-152, Jackson ImmunoResearch, USA), and used at 1/250 dilution for 45 min at room temperature. DAPI (S2110, Solarbio, China) was applied to stain the cell nucleus for 5 min. Immunofluorescence-stained cells were examined under a laser-scanning confocal microscope (Nikon, Japan).

### Western Blot

Cell lysates were prepared in radio immunoprecipitation assay lysis buffer with fresh protease and phosphatase inhibitors mixture. Protein concentrations were measured by BCA protein assay reagent (P0006, Beyotime Biotechnology, China). In brief, protein (20 μg) was resolved by sodium dodecyl sulfate–polyacrylamide gel electrophoresis and transferred to polyvinylidene fluoride membranes. After blocking in tris-buffered saline tween (TBST) buffer with 5% nonfat milk for 1 h, the membranes were incubated with the following primary antibodies: glutathione peroxidase4 (GPX4) (rabbit, 17 kDa, ab125066, 1:5,000 dilution, Abcam), 4-hydroxynonenal (4-HNE) (rabbit, 17–76 kDa, ab46545, 1:3,000 dilution, Abcam), 12/15-LOX (rabbit, ∼75 kDa, BS8547, 1:1,000 dilution, Bioworld), and β-actin (mouse, 43 kDa, A5441, 1:5,000 dilution, Sigma), overnight at 4°C. The next day, after being washed in TBST buffer three times, the membranes were further incubated with secondary antibodies (mouse, A9044, 1:10,000 dilution, Sigma; rabbit, A9169, 1:10,000 dilution, Sigma). Immunoreactive bands were visualized by the ChemiDoc XRS+ imaging system (Bio-Rad, USA). β-Actin was used as an internal control. For quantification of the protein bands, we utilized Image J software (National Institutes of Health, USA) to obtain densitometric values.

### Statistical Analysis

Results were expressed as mean ± SD. Statistical signiﬁcance was determined by analysis of variance (ANOVA) followed by a multiple comparison test with a Bonferroni test. P-values of less than 0.05 were considered statistically signiﬁcant.

## Results

### Baicalein Attenuates FeCl_3_-Induced Epileptic Seizure Behavior

**Figure 1:**
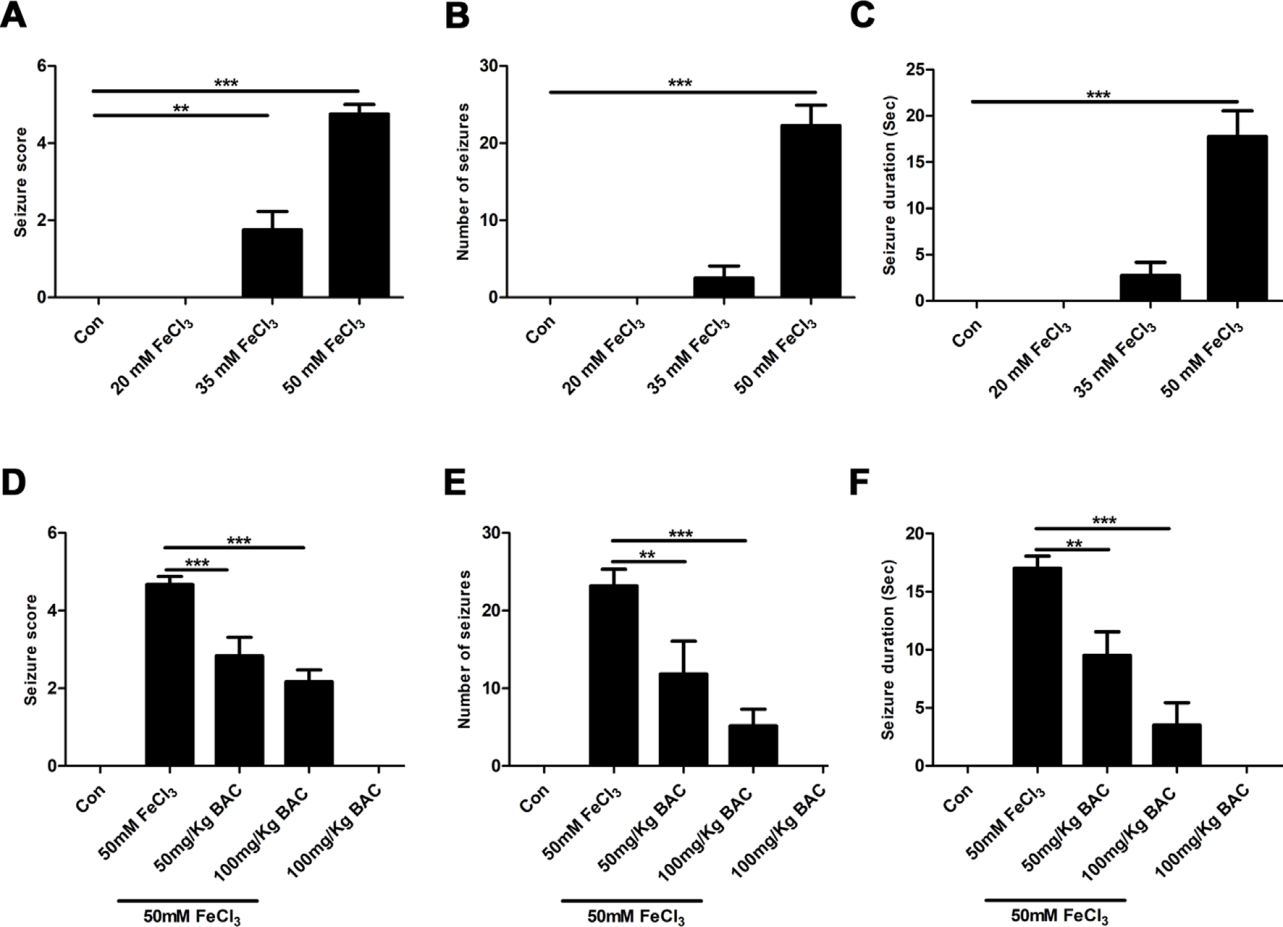
Baicalein inhibits FeCl_3_-induced behavioral seizures. **(A–C)** Behavioral seizures induced by different concentrations of FeCl_3_ (20, 35, and 50 mM) were presented as seizure score, number of seizures, and average seizure duration (Sec), n = 6. **(D–F)** Intraperitoneal injection of different doses of baicalein (50 and 100 mg/kg) 30 min prior to the injection of 50 mM FeCl_3_. The seizure score, number of seizures, and average seizure duration (Sec) were reduced significantly at different doses of baicalein, n = 6. *p < 0.05, **p < 0.01, and ***p < 0.001.

We first examined whether baicalein could attenuate epileptic seizure behavior in the FeCl_3_-induced PTE model. We used four different concentrations (20, 35, 50, 100 mM) of FeCl_3_ to investigate the FeCl_3_-induced PTE model. Mice with grade 4–5 seizures were considered successful. It was found that an administration of a dose of 50 mM FeCl_3_ or 100 mM FeCl_3_ (data not shown) resulted in increasing convulsive activity, leading to generalized tonic–clonic seizure (**Figure 1A**). And we also observed that the number and duration time of seizure were significantly elevated with the increase in dose of FeCl_3_ (**Figure 1B and C**). However, the mortality rate of mice was high in the 100 mM FeCl_3_ injection group (data not shown). So, we performed the FeCl_3_-induced PTE model by injecting 5 μl of 50 mM FeCl_3_ into the sensorimotor cortex of the mice. All of the mice in each group survived without any complications at the end of the experiment period. We then pretreated with different concentrations of baicalein (50 and 100 mg/kg) before administration of a dose of 50 mM FeCl_3_. The data showed that preadministration of 100 mg/kg baicalein significantly reduced the seizure score, number of seizures, and seizure duration compared to the FeCl_3_-induced posttraumatic epileptic seizure group (**Figure 1D–F**). Moreover, the 100 mg/kg dose of baicalein had no toxic effect on sham control mice.

### Baicalein Alleviates FAC-Induced Neuronal Damage by Inhibiting Ferroptosis

**Figure 2:**
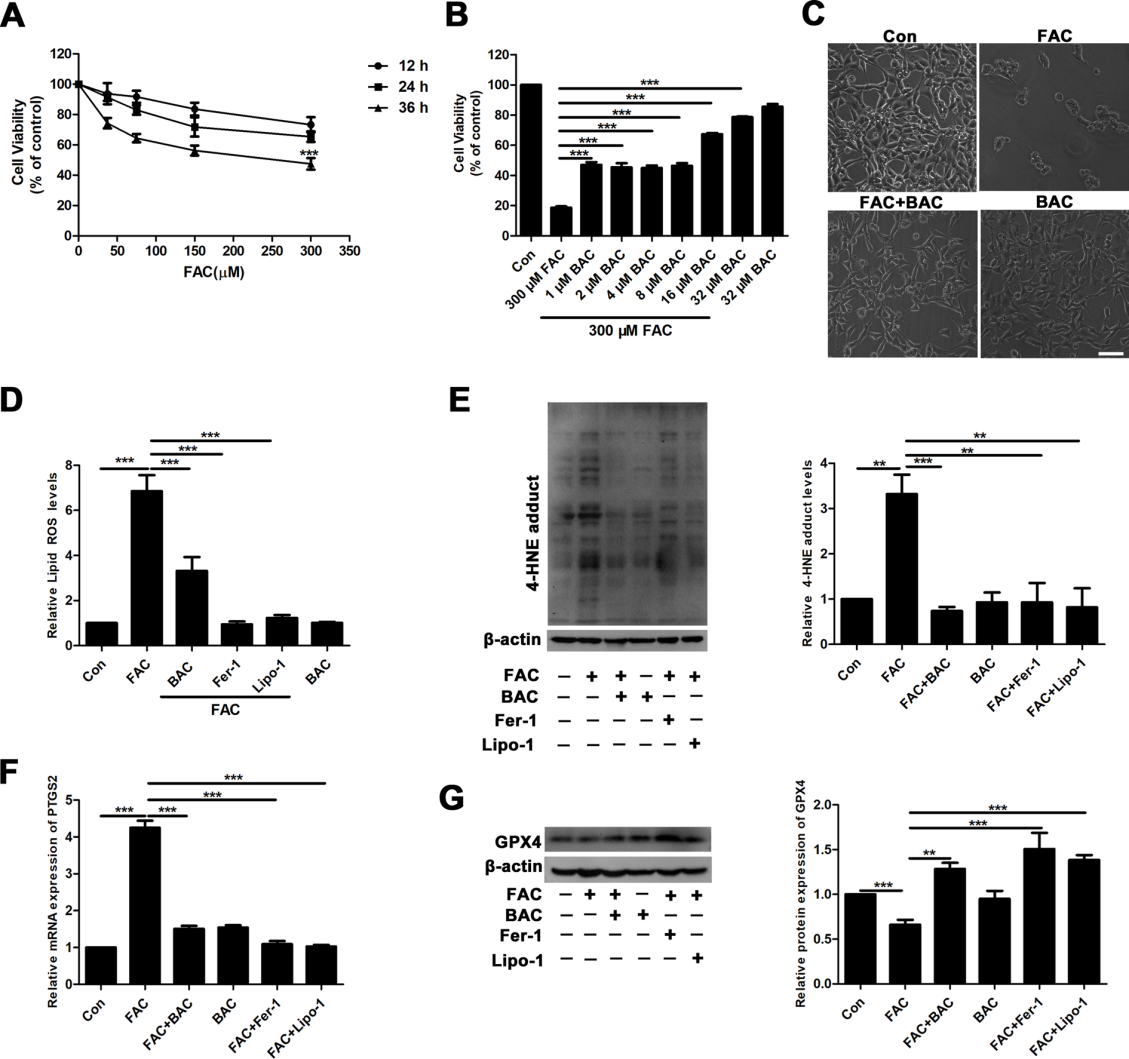
Baicalein attenuates FAC-induced neuronal damage by inhibiting ferroptosis. **(A)** Cell viability was measured with CCK8 assay, in which HT22 cells were incubated with various concentrations (37.5, 75, 150, and 300 μM) of FAC for different periods of time (12–36 h). **(B)** HT22 cells were incubated with different concentrations (1, 2, 4, 8, 16, and 32 μM) of baicalein for 2 h, prior to incubation with 300 μM of FAC for 36 h. **(C)** FAC exposure caused cell shrinkage and nuclear condensation. Baicalein pretreatment reduced cell death. Scale bar: 200 μm **(D)** after 2 h pretreatment with 32 μM baicalein, 12.5 μM Fer-1, and 1 μM Lipo-1, lipid ROS in HT22 cells was assessed by FACS analysis of C11-BODIPY ﬂuorescence after treatment with FAC for 36 h. **(E)** Representative Western blots of 4-HNE with 32 μM baicalein and ferroptosis inhibitors pretreatment for 2 h in the FAC-induced HT22 cell injury model. **(F)** The relative mRNA expression of PTGS2 was reduced after baicalein and ferroptosis inhibitors pretreatment. **(G)** Representative Western blots of GPX4 with 32 μM baicalein and ferroptosis inhibitors pretreatment for 2 h in the FAC-induced HT22 cell injury model. Data are representative of three independent experiments with similar results, *p < 0.05, **p < 0.01, and ***p < 0.001.

**Figure 3:**
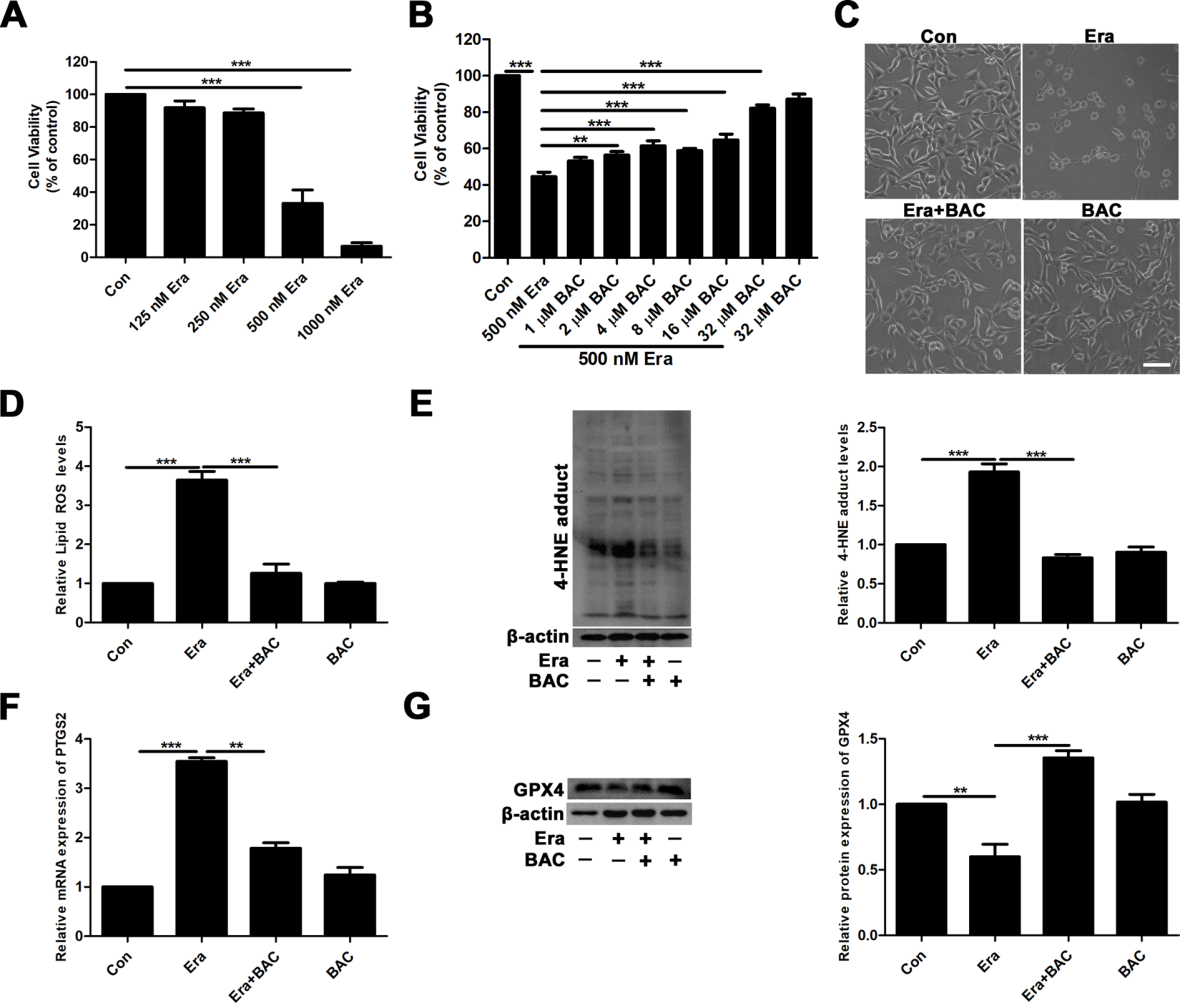
Baicalein attenuates erastin-induced neuronal damage by inhibiting ferroptosis. **(A)** Cell viability was measured with CCK8 assay, in which HT22 cells were incubated with various concentrations (125, 250, 500, and 1,000 nM) of erastin for 8 h. **(B)** HT22 cells were incubated with different concentrations of baicalein for 2 h, prior to incubation with 500 nM erastin for 8 h. **(C)** Baicalein (32 μM) pretreatment could reduce cell death induced by 500 nM erastin. Scale bar: 200 μm **(D)** after 2 h pretreatment with 32 μM baicalein, lipid ROS in HT22 cells was assessed by FACS analysis of C11-BODIPY ﬂuorescence after treatment with erastin for 8 h. **(E)** Representative Western blots of 4-HNE with 32 μM baicalein pretreatment for 2 h in the erastin-induced HT22 cell injury model. **(F)** The relative mRNA expression of PTGS2 was reduced after baicalein pretreatment. **(G)** Western blots illustrated that GPX4 was significantly diminished in the erastin-induced HT22 cell injury model and baicalein treatment reversed this phenomenon. Data are representative of three independent experiments with similar results, *p < 0.05, **p < 0.01, and ***p < 0.001.

To further elucidate the potential neuroprotective mechanism of baicalein against posttraumatic epileptic seizures, we then established an *in vitro* iron-induced neuronal injury model using FeCl_3_. However, there was no significant effect after intervention with different concentrations of FeCl_3_ in HT22 cells (**Supplementary Figure S1**). Alternatively, we used HT22 cells with FAC as a source of iron according to the previous descriptions (Lee et al., 2016; Lee et al., 2018). The toxic effects of FAC on HT22 cells were detected *via* CCK-8 assay. It was obviously found that FAC at a concentration of 300 μM for 36 h exerted the most potent impairment on HT22 hippocampal neurons (nearly 50% reduction in comparison with vehicle control), as indicated in **Figure 2A**. The cytotoxic effect was attenuated by pretreatment with baicalein for 2 h in a dose-dependent manner (**Figure 2B**). It was noteworthy that baicalein at a concentration of 32 μM exerted the most obvious protective effects. Morphologically, it was also found that 32 μM baicalein improved cell viability the most in HT22 cells under FAC exposure (**Figure 2C)**. Furthermore, in HT22 cells following FAC treatment, baicalein (32 μM) robustly suppressed ferroptotic indices, including lipid ROS, 4-HNE (a common lipid peroxidation by-product) levels, and the relative mRNA expression of PTGS2 (a downstream marker of ferroptosis), as compared with treatment with Fer-1 or Lipo-1 (**Figure 2D–F**). Additionally, we also found that baicalein could significantly upregulate the expression of GPX4 (a crucial regulator of ferroptosis) (**Figure 2G**). The above-mentioned results were also validated in HT22 neurons after exposure of erastin (a specific ferroptotic inducer) exposure (**Figure 3A–G**).

### 12/15-LOX is Involved in the Inhibition of Ferroptosis in FAC-Induced HT22 Cell Injury Model by Baicalein

**Figure 4:**
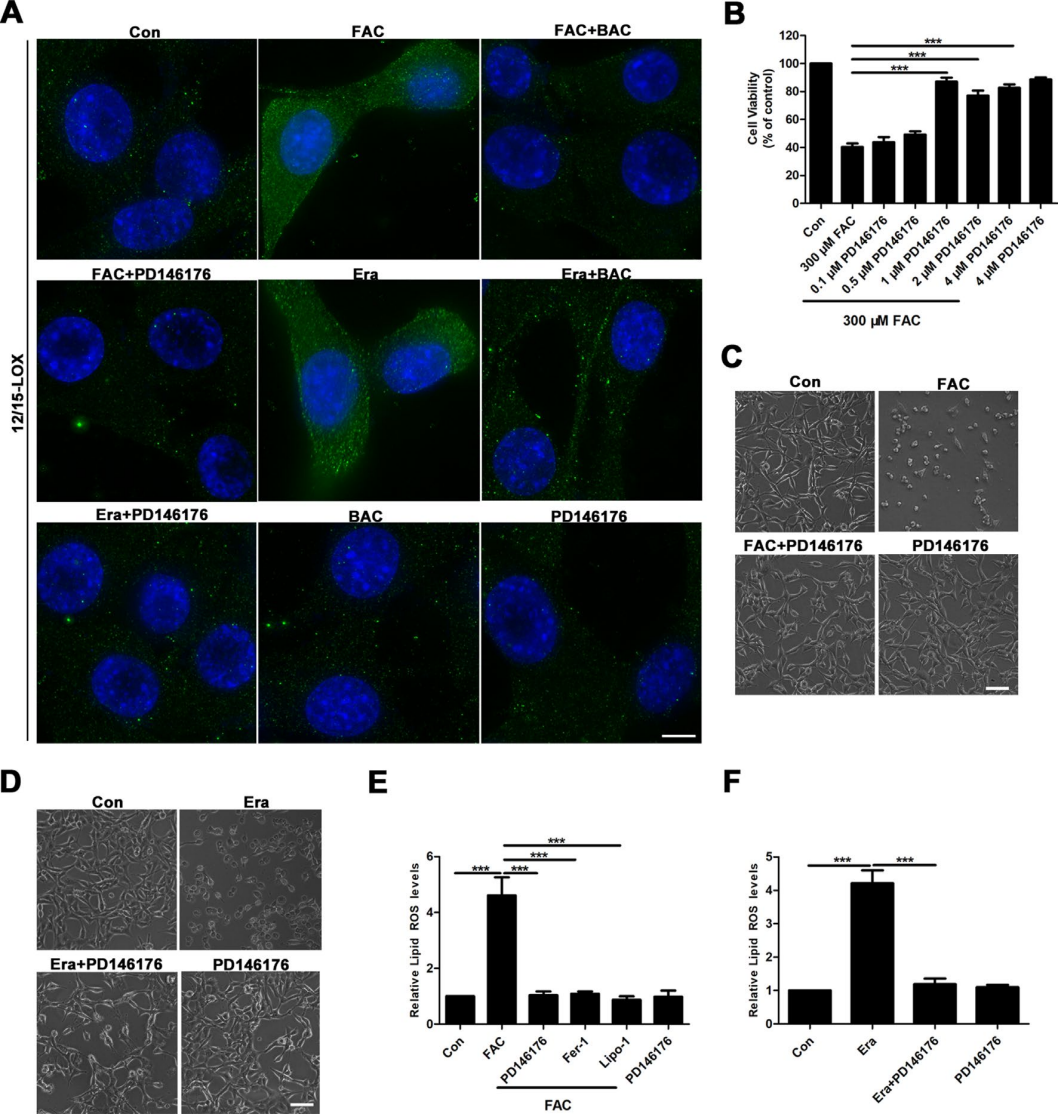
12/15-LOX is involved in the inhibition of ferroptosis in FAC-induced HT22 cell injury model by baicalein. **(A)** Immunofluorescence analysis indicated that FAC and erastin significantly increased the expression of 12/15-LOX in HT22 cells, which was reversed by baicalein and PD146176. Scale bar: 50 μm **(B)** CCK8 analysis showed that pretreatment with PD146176 could reverse cell damage induced by FAC. **(C–D)** Exposure to FAC and erastin caused HT22 cells to shrink, and 1 μM PD146176 reversed this phenomenon. Scale bar: 200 μm **(E–F)** pretreatment with 1 μM PD14617, 12.5 μM Fer-1, and 1 μM Lipo-1 could reverse increased lipid ROS in HT22 cells induced by FAC and erastin. Data are representative of three independent experiments with similar results, *p < 0.05, **p < 0.01, and ***p < 0.001.

Given that baicalein was reported to be a selective inhibitor of 12/15-LOX (Deschamps et al., 2006; Li et al., 2019), we next investigated whether or not 12/15-LOX was involved in ferroptosis in the FAC-induced HT22 cell damage model. To achieve this, we analyzed expression levels of 12/15-LOX in the FAC- or erastin-induced cell impairment model by immunofluorescence. Compared with the FAC or erastin group, significantly decreased immunofluorescence signals for 12/15-LOX were present in the baicalein pretreatment group, which was consistent with the effect of PD146176 (a specific 12/15-LOX inhibitor) on significantly reducing the immunofluorescence signals of 12/15-LOX (**Figure 4A**). Meanwhile, as shown in **Figure 4B**, 1 μM PD146176 remarkably reversed FAC-induced cell damage. Consistently, we observed that pretreatment with PD146176 could alleviate FAC- and erastin-induced cell damage in morphology (**Figure 4C and D**). Additionally, we also found that pretreatment with PD146176, Fer-1, and Lipo-1 remarkably suppressed the production of lipid ROS induced by FAC and erastin (**Figure 4E and F**). The above results indicated that baicalein-mediated inhibition of 12/15-LOX contributed to the inhibition of FAC-induced ferroptosis.

### Baicalein Counteracts FeCl_3_-Induced Epileptic Seizures by Inhibiting Ferroptosis

**Figure 5:**
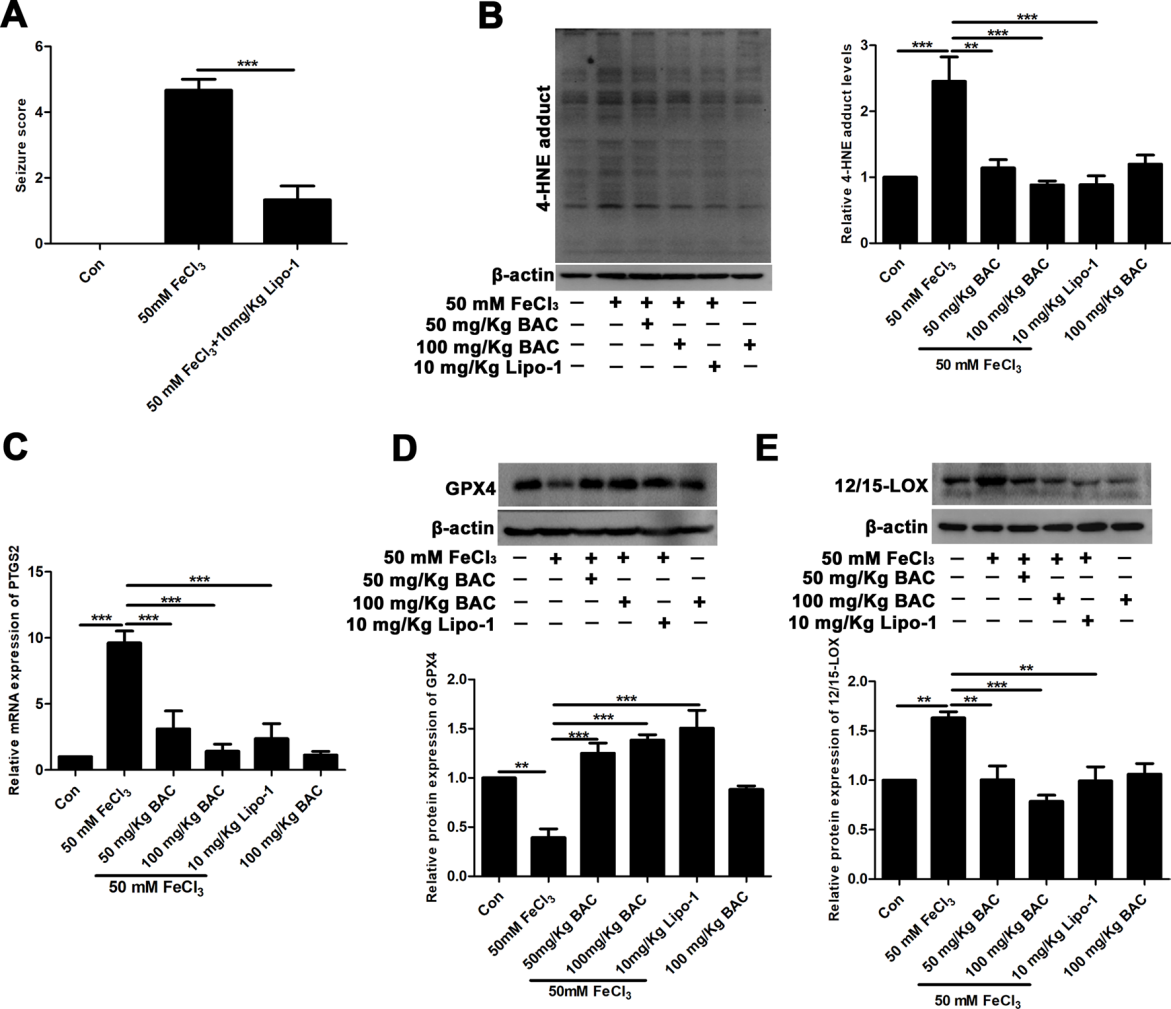
Baicalein counteracts FeCl_3_-induced epileptic seizure behavior by inhibiting ferroptosis. **(A)** Decreased seizure score after treatment with Lipo-1 (a specific inhibitor of ferroptosis) in the FeCl_3_-induced PTE model. **(B)** Western blots showing expression of 4-HNE in hippocampus tissues of FeCl_3_-induced PTE mice with different doses of baicalein and Lipo-1 pretreatment, n = 6. **(C)** The relative mRNA expression of PTGS2 was reduced after baicalein and Lipo-1 pretreatment, n = 6. **(D)** Western blots illustrated that GPX4 was significantly diminished in FeCl_3_-induced PTE mice, and baicalein and Lipo-1 treatment reversed this phenomenon (n = 6 for both groups). **(E)** Baicalein inhibited the increase of 12/15-LOX in hippocampus tissues from FeCl_3_-induced PTE mice, n = 6. *p < 0.05, **p < 0.01, and ***p < 0.001.

Next, we validated the results of the *in vitro* experiments in the FeCl_3_-induced seizure model. The results of reduced production of 4-HNE and lipid ROS after pretreatment of baicalein and ferroptosis inhibitors suggested that ferroptosis was involved in the neuroprotective effects of baicalein. Lipo-1, a fat-soluble small molecule compound, is a specific inhibitor of ferroptosis. So, we evaluated the effects of Lipo-1 in the FeCl_3_-induced PTE seizure model. Lipo-1 was administered to mice at a dose of 10 mg/kg daily for 3 days prior to administration of FeCl_3_. Similar to the previous results, we found that Lipo-1 could significantly reduce the seizure score of mice in the FeCl_3_-induced PTE seizure model (**Figure 5A**). We next collected hippocampus tissue from those mice and compared the levels of the production of 4-HNE and the mRNA expression of PTGS2. As **Figure 5B and C** shows, in the FeCl_3_-induced PTE seizure model, the production of 4-HNE and the mRNA expression of PTGS2 was significantly diminished after pretreatment with baicalein and Lipo-1 compared to the group treatment with FeCl_3_ alone. In addition, we also examined the changes of GPX4. It was found that the expression of GPX4 was significantly down-regulated, but this phenomenon was reversed when pretreated with baicalein and Lipo-1 (**Figure 5D**). Consistent with the *in vitro* results, expression of 12/15-LOX was increased in hippocampus from the FeCl_3_-induced PTE seizure model, and pretreatment with baicalein remarkably inhibited the increase of 12/15-LOX (**Figure 5E**). Overall, the results that pretreatment with baicalein and Lipo-1 could reduce the production of 4-HNE, the mRNA expression of PTGS2, and the expression of 12/15-LOX and up-regulate the expression of GPX4 in FeCl_3_-induced posttraumatic epileptic seizures further support that baicalein could exert neuroprotective effects in FeCl_3_-induced epileptic seizures by inhibiting 12/15-LOX-related ferroptosis.

## Discussion

Our study showed that pretreatment with baicalein in FeCl_3_-induced epileptic seizure mice significantly improved the epileptic seizure behavior and exhibited great neuroprotection in PTE mice. In particular, baicalein exerted neuroprotective effects in PTE mice by inhibiting ferroptosis, and inhibition of 12/15-LOX was related to its antiferroptosis activity. More importantly, these results provide a new therapeutic drug for the treatment of PTE.

With the wide application of neuroprotective strategies in neurological disease (Szwajgier et al., 2017; Mendes et al., 2019), traditional Chinese medicine has attracted increasing attention due to its advantages of easy penetration of blood–brain barrier and abundance in nature (Zeng, 2017; Chen and Lin, 2018). Baicalein (5,6,7-trihydroxyflavone) is a type of flavonoid compound, which can be acquired from the roots of *Scutellaria baicalensis*, has possessed remarkable neuroprotective effects in neurological diseases, such as AD and PD (Li et al., 2011; Gao et al., 2015; Gu et al., 2016; Zhou et al., 2016; Dinda et al., 2017; Liu et al., 2017). In addition, it has recently been reported that baicalein could exert significant neuroprotective effects in the treatment of temporal lobe epilepsy by reducing oxidative stress and inflammation (Qian et al., 2019). And our previous study suggested that baicalein could reduce the total time and number of epileptic discharges in the inherited epilepsy model *via* suppressing oxidative stress (Mao et al., 2014). Our present extensive work further disclosed the obvious neuroprotection against FeCl_3_-induced posttraumatic epileptic seizures, a kind of important acquired epileptic seizure and this neuroprotective mechanism may be related to suppressing 12/15-LOX-involved ferroptosis. Posttraumatic epileptic seizures were previously reported to be successfully established by FeCl_3_ (Pitkanen and McIntosh, 2006). Currently, our present work firstly prepared the mouse model of posttraumatic epileptic seizures using different doses of FeCl_3_, and we found that FeCl_3_ by the dose of 50 mM was sufficient for inducing posttraumatic epileptic seizures in mice.

**Figure 6:**
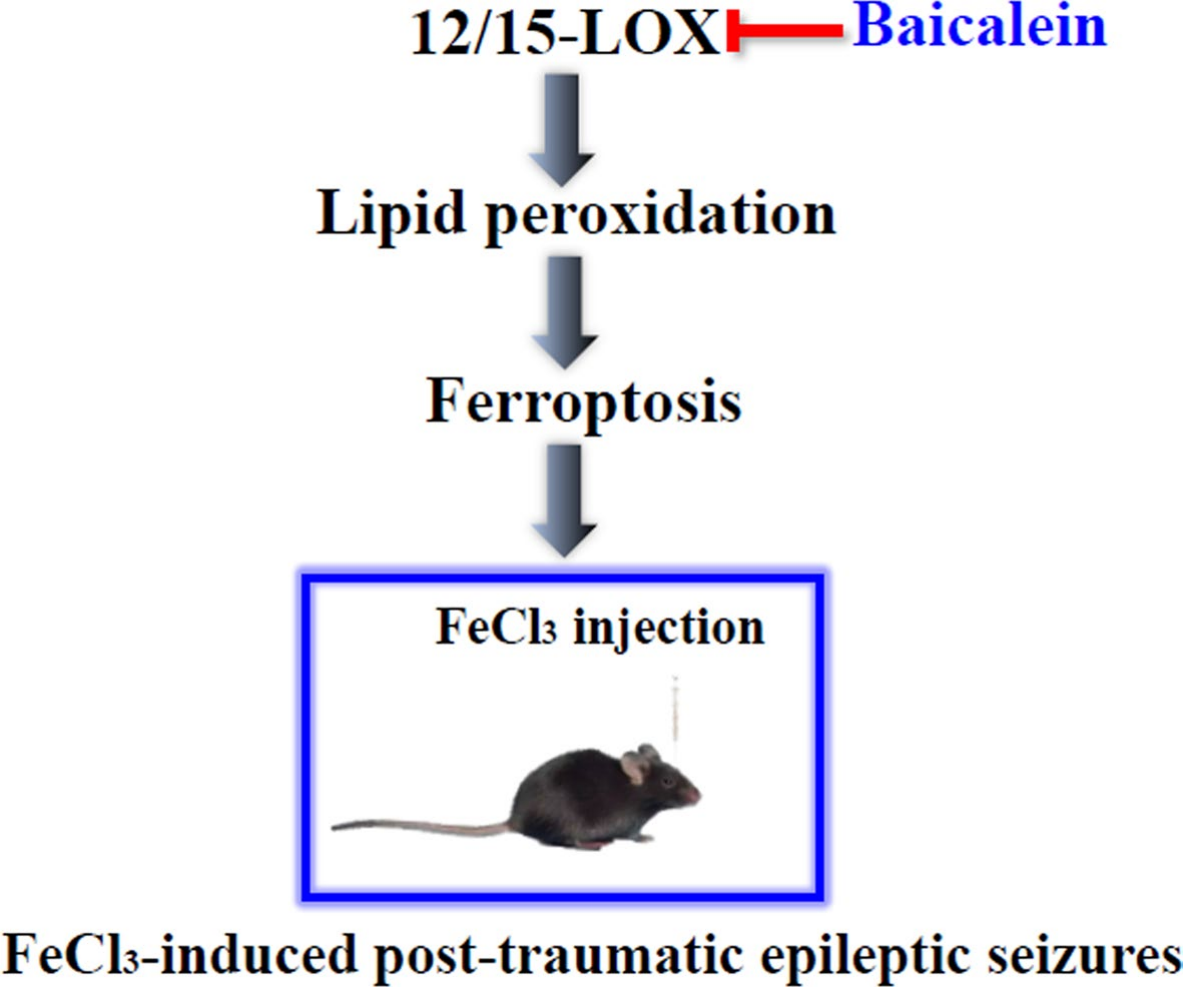
The graph depicted the neuroprotective role of baicalein in FeCl_3_-induced posttraumatic epileptic seizures. Baicalein ameliorated the FeCl_3_-induced posttraumatic epileptic seizures by inhibiting 12/15-LOX-associated ferroptosis.

We next focused on the neuroprotective mechanisms of baicalein in posttraumatic epileptic seizures. According to previous reports, the neuroprotective effects of baicalein are closely correlated with its antioxidant properties (Gao et al., 2015; Gu et al., 2016; Zhou et al., 2016). Oxidative stress is intricately involved in the pathogenesis of a variety of neurological diseases, including epilepsy, AD, PD, cerebral ischemia–reperfusion injury, etc. (Butterfield and Lauderback, 2002; Wang et al., 2011; Carvalho et al., 2017; Pauletti et al., 2017; Pearson-Smith and Patel, 2017; Su et al., 2017; Cheignon et al., 2018; Deng et al., 2018; He et al., 2018; Xie et al., 2018b; Xiao et al., 2019). Previous studies indicated that the brain was particularly sensitive to lipid peroxidation-induced damage due to high consumption of oxygen and abundant PUFAs (Salim, 2017). Therefore, lipid peroxidation is the main form of neuronal oxidative damage. Indeed, lipid peroxidation is thought to be involved in the pathogenesis of PTE (Srivastava et al., 2019). Ferroptosis is a lipid peroxidation-dependent new form of oxidative cell death (Dixon et al., 2012; Mao et al., 2018), and can be inhibited by iron chelators and lipid peroxidation inhibitors (e.g., Fer-1 and Lipo-1). And we found that ferroptosis was involved in the neuroprotective effects of baicalein in FeCl_3_-induced PTE seizures. To investigate the underlying neuroprotective mechanisms of baicalein *in vitro*, we established an iron-overloaded HT22 cell damage model by using FAC as a source of iron, but not FeCl_3_. This was because FeCl_3_ failed to induce HT22 cell damage (**Supplementary Figure S1**). FAC is a trivalent organic iron salt used as an iron fortifier for food in the treatment of iron deficiency anemia. It can be absorbed after being reduced to ferrous iron in the body. Moreover, the absorption effect is superior to that of inorganic iron (FeCl_3_) (Lee et al., 2016; Lee et al., 2018). In the FAC-induced HT22 cell damage model, baicalein could reduce the increase of 4-HNE and lipid ROS induced by FAC. Consistent results were obtained in the erastin-induced HT22 cell injury model. This indicated that ferroptosis was involved in FAC-induced HT22 cell damage, and baicalein might act as an inhibitor of lipid peroxidation to suppress ferroptosis. In addition to antioxidant actions, baicalein was reported to restrain the function of 12/15-LOX (Deschamps et al., 2006; Li et al., 2019). 12/15-LOX is an enzyme that oxidizes PUFAs to produce many bioactive lipid metabolites, and is also an important regulator of ferroptosis (Probst et al., 2017; Kenny et al., 2019). Numerous studies have demonstrated its involvement in the pathogenesis of human diseases (Singh and Rao, 2019). In addition, mice lacking leukocyte-type 12/15-LOX were resistant to PTZ-induced epilepsy, which implied that 12/15-LOX might be involved in the process of epileptic seizures (Kanzler et al., 2018). In our study, the expression of 12/15-LOX was increased in the FAC- and erastin-treated HT22 cells and FeCl_3_-induced mice PTE model. Pretreatment with baicalein dramatically inhibited the increase of 12/15-LOX. Intervention of PD146176 (a specific inhibitor of 12/15-LOX) also significantly reduced the production of lipid ROS induced by FAC and erastin. These findings indicated that baicalein-mediated inhibition of 12/15-LOX contributed to its antiferroptosis activity. Ferroptosis was also reported to take part in glutamate-induced death in a rat organotypic hippocampal slice culture model. The findings revealed that ferroptosis was indeed involved in brain diseases (Dixon et al., 2012). Additionally, ferroptosis was closely associated with neurodegenerative diseases, and could affect the occurrence and development of PD and AD by regulating lipid peroxidation (Xie et al., 2016; Yang and Stockwell, 2016). Moreover, the potent neuroprotective effects of ferroptosis inhibitors in various brain diseases, such as some forms of traumatic and hemorrhagic brain damage, implied that ferroptosis might constitute a potential target for brain disorder therapy (Dixon et al., 2012; Skouta et al., 2014; Do Van et al., 2016; Stockwell et al., 2017; Zille et al., 2017; Anthonymuthu et al., 2018; Xie et al., 2018a; Zhang et al., 2018b). In fact, in the kainic acid-induced rat epilepsy model, it was found that ferroptosis was involved in the pathogenesis of cognitive dysfunction in temporal lobe epilepsy and Fer-1 could improve cognitive dysfunction and hippocampal neuron loss in epileptic rats (Ye et al., 2019). In our present investigation, we found that ferroptosis was involved in the development of posttraumatic epileptic seizures and Lipo-1 could remarkably suppress behavioral seizures in FeCl_3_-induced seizures in mice. Our studies further demonstrated that baicalein protected neurons against ferroptotic cell death in seizures induced by FeCl_3_ and 12/15-LOX was involved in baicalein’s neuroprotection (**Figure 6**).

In summary, our study revealed that ferroptosis was involved in the pathogenesis of PTE. Baicalein, as a naturel bioactive compound, could ameliorate behavioral seizures and play a key neuroprotective role in FeCl_3_-induced posttraumatic epileptic seizures through inhibiting ferroptosis and its neuroprotection might be related to suppression of 12/15-LOX. Our work further extended the neuroprotective action of baicalein in posttraumatic epileptic seizures and provided a theoretical basis for its potential clinical translational applications in PTE.

## Data Availability Statement

The raw data supporting the conclusions of this manuscript will be made available by the authors, without undue reservation, to any qualified researcher.

## Ethics Statement

The protocol of animal experiment was approved by the Medical Ethics Committee of Xiangya Hospital and performed in accordance with the National Institute of Health Guide for the Care and Use of Laboratory Animals. All efforts had been made to minimize the animal’s suffering.

## Author Contributions

X-YM conceived and designed the study. QL, Q-QL, J-NJ, and Q-YS performed the experiments. X-YM and H-HZ provided experimental support. QL wrote the paper. X-YM and W-LJ revised the manuscript.

## Funding

This work is partially financially supported by the National Natural Science Foundation of China (nos. 81671293 and 81302750), Natural Science Foundation of Hunan Province (no. 2017JJ3479), the Fundamental Research Funds for the Central Universities of Central South University (2018zzts900), and the Hunan Provincial Department of Education Innovation Platform Open Fund Project (no. 17K100).

## Conflicts of Interest Statement

The authors declare that the research was conducted in the absence of any commercial or financial relationships that could be construed as a potential conflict of interest.

## Abbreviations

PTE, posttraumatic epilepsy; TBI, traumatic brain injury; AD, Alzheimer’s disease; PD, Parkinson’s disease; TCM, traditional Chinese medicine; PUFAs, polyunsaturated fatty acids; 12/15-LOX, 12/15-lipoxygenase; DMEM, Dulbecco’s modiﬁed Eagle’s medium; FBS, fetal bovine serum; BAC, baicalein; FAC, ferric ammonium citrate; FeCl_3_, iron chloride; Fer-1, ferrostatin-1; Lipo-1, liproxstatin-1; CCK8, cell counting kit-8; PBS, phosphate buffer saline; HBSS, Hanks’ balanced salt solution; TBST, tris-buffered saline tween; PTGS2, prostaglandin endoperoxide synthase 2; Lipid ROS, lipid reactive oxygen species; MDA, malondialdehyde; 4-HNE, 4-hydroxynonenal; GPX4, glutathione peroxidase 4.

## Supplementary Material

Figure S1 Iron overload induces neuronal death in HT22 cells. HT22 cells were incubated with different concentrations of FAC and FeCl_3_ (37.5 μM, 75 μM, 150 μM, 300 μM, 600 μM) for 36 h. Cell morphology was observed by an inverted microscope.
