# Supplementary figures and images for: Baicalein Exerts Neuroprotective Effects in FeCl3-Induced Posttraumatic Epileptic Seizures via Suppressing Ferroptosis
Source: Front Pharmacol. 2019 Jun 7;10:638. doi: 10.3389/fphar.2019.00638 (PMC6568039; doi:10.3389/fphar.2019.00638)

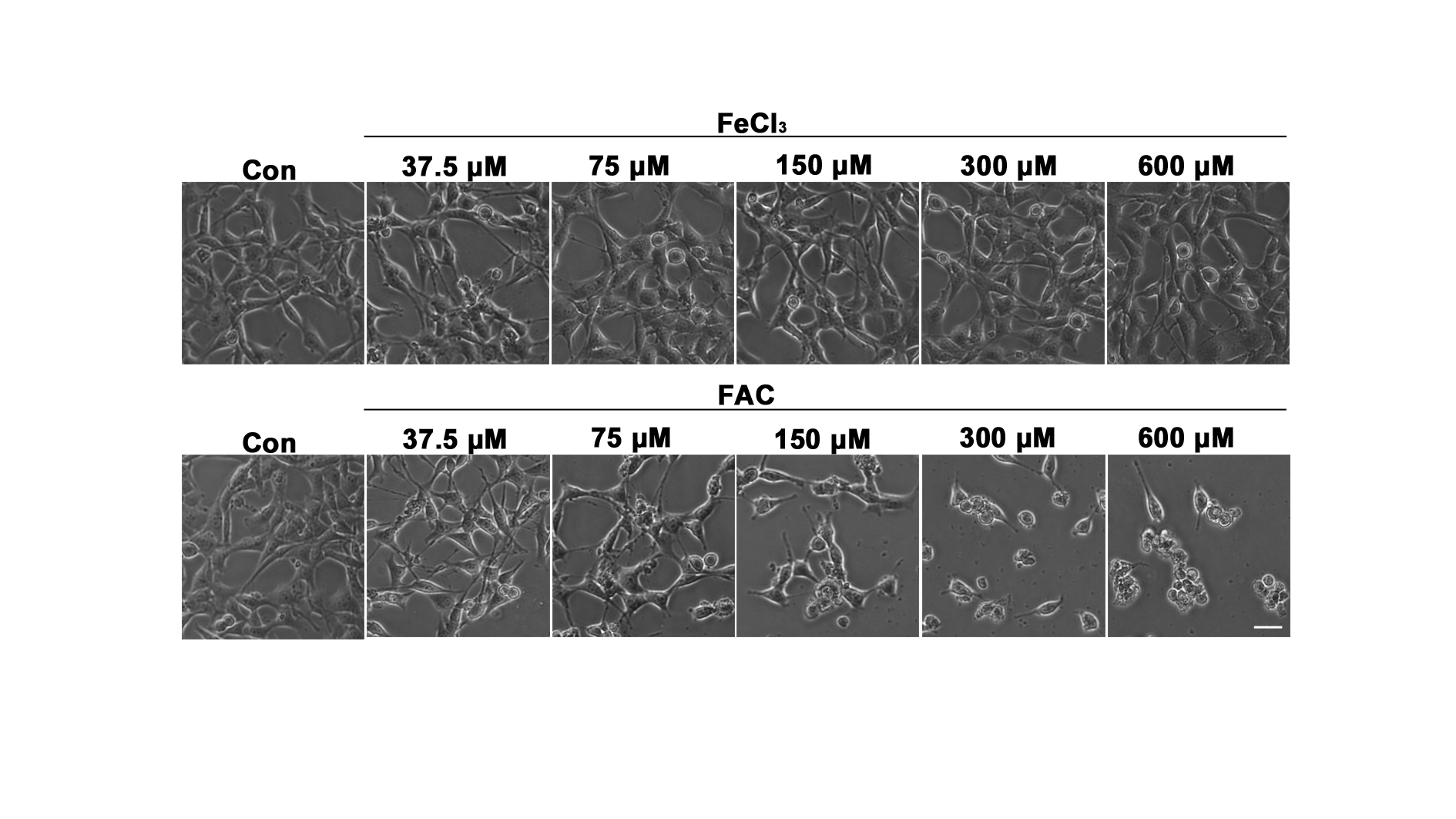

Supplement: Figure S1 — Iron overload induces neuronal death in HT22 cells. HT22 cells were incubated with different concentrations of FAC and FeCl3 (37.5 μM, 75 μM, 150 μM, 300 μM, 600 μM) for 36 h. Cell morphology was observed by an inverted microscope. [file Image_1.tif]
